# Nonlinear phenomena in models of the circadian clock

**DOI:** 10.1098/rsif.2020.0556

**Published:** 2020-09-30

**Authors:** Inge van Soest, Marta del Olmo, Christoph Schmal, Hanspeter Herzel

**Affiliations:** 1 Institute for Theoretical Biology, Charité and Humboldt Universität zu Berlin, 10115 Berlin, Germany; 2 Master Program Neuroscience and Cognition, Utrecht University, Utrecht, The Netherlands

**Keywords:** circadian clock, mathematical modelling, bifurcation, nonlinear phenomena, feedback regulation

## Abstract

The mammalian circadian clock is well-known to be important for our sleep–wake cycles, as well as other daily rhythms such as temperature regulation, hormone release or feeding–fasting cycles. Under normal conditions, these daily cyclic events follow 24 h limit cycle oscillations, but under some circumstances, more complex nonlinear phenomena, such as the emergence of chaos, or the splitting of physiological dynamics into oscillations with two different periods, can be observed. These nonlinear events have been described at the organismic and tissue level, but whether they occur at the cellular level is still unknown. Our results show that period-doubling, chaos and splitting appear in different models of the mammalian circadian clock with interlocked feedback loops and in the absence of external forcing. We find that changes in the degradation of clock genes and proteins greatly alter the dynamics of the system and can induce complex nonlinear events. Our findings highlight the role of degradation rates in determining the oscillatory behaviour of clock components, and can contribute to the understanding of molecular mechanisms of circadian dysregulation.

## 1. Introduction

Circadian clocks are important in the coordination of physiological rhythms in many organisms including cyanobacteria, fungi, plants, insects and mammals [1,2]. The period of the circadian clock is approximately 24 h [3], but ranges from 19 to 20 h in some spider species [4] to more than 24 h in humans [5,6]. Importantly, the circadian clock can be synchronized to external Zeitgebers, such as light–dark and temperature cycles. The resulting entrainment provides evolutionary advantages, for example by enabling organisms to adapt to the 24 h day–night rhythm [7,8]. Molecular circadian rhythms are generated by gene-regulatory feedback loops [9,10], which are even observed in single cells [11,12]. Such single cell circadian oscillators are organized into tissue networks which are then hierarchically arranged to constitute the mammalian circadian timing system.

Mammalian circadian clocks can be regarded as a system of coupled nonlinear oscillators. It is well-known from the theory of nonlinear dynamics that coupled oscillators can generate limit cycles, tori, period-doubling and even chaos [13–15]. Although, strictly speaking, limit cycles are inherently nonlinear phenomena, we use this term to refer to more complex dynamics, namely toroidal oscillations, period-doubling and chaotic dynamics. The quantification of complex nonlinear phenomena, such as beating envelopes or chaotic oscillations, is difficult in chronobiology because of the need of long-term recordings, which are a limitation in most experimental setups. Thus, in the absence of accurate long-term and stationary recordings, it is challenging to apply established attractor theory and analysis [16–20]. Most circadian clock models have focused on the mechanisms of rhythm generation [21–25], on synchronization [26,27] and on entrainment to Zeitgeber signals [28–32]. Only a few modelling studies have been devoted to characterize experimentally observed tori, period-doubling and chaos, yet still in the presence of external forcing [28,32–35].

**Figure 1.**
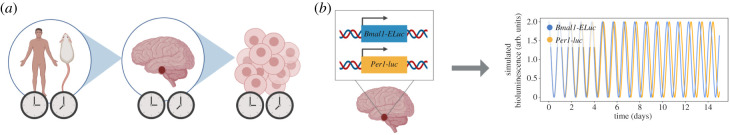
Dissociation or ‘splitting’ of circadian rhythms. (*a*) Dissociation of circadian rhythms, indicated by the two clocks, has been shown to occur under some circumstances both at the organismic and tissue level [2,3,33,36–40], but still requires validation at the molecular level. (*b*) Evidence for rhythm dissociation in mouse neonatal SCN. Simulated sine waves with the period values obtained from *Bmal1-ELuc* and *Per1-luc* rhythms in mouse neonatal SCN harbouring two reporters, namely 22.7 h (*Bmal1-Eluc*) and 23.1 h (*Per1-luc*) [39]. Reporter rhythms have been simulated to appear initially in phase, so that the period difference becomes more evident.

On the organismic level, chronobiological research has revealed certain complex nonlinear phenomena in unforced systems. Some examples are the dissociation of activity rhythms from oscillations in melatonin, body temperature and urine production [2,6,36], or the coexistence of two periods in behavioural activity recordings, which is termed ‘splitting’ in the literature (figure 1*a*) [33,37,38]. Nevertheless, complex nonlinear phenomena at the molecular level in mammalian circadian clocks have only been suggested. Experimental and computational studies have shown that rhythmic clock reporter signals, which apparently should have the same period over time, dissociate in the SCN at least transiently (figure 1*b*) [31,39,40]. Moreover, it has been suggested that the divergent rhythms might be traced back to single cells, in which different feedback loops could be responsible for the distinct periods [31,39]. The observations on splitting, however, still require validation, ideally with double-transgenic cellular systems harbouring two reporters of different colours.

At the single cell level, the molecular circadian clock is explained by auto-regulatory transcription–translation feedback loops, in which protein products regulate the transcription of their own genes, either alone or in combination with other clock proteins. The clock proteins that were initially found as primary generators of mammalian circadian rhythms were BMAL1, CLOCK, PERs and CRYs [10]. BMAL1 and CLOCK are usually defined as activators that induce the transcription of PERs and CRYs, while these proteins in turn repress the CLOCK : BMAL1 complex and thus establish negative feedback loops [9,10]. A number of studies have focused on the roles of CLOCK : BMAL1 and PER : CRY in the generation of oscillations. Nevertheless, in the last decades, additional core clock proteins have been identified such as REVERBs, RORs and E4BP4, among others. These clock proteins interact with each other by different positive and negative feedback loops, including the BMAL1-REVERB loop in addition to the well-known PER-CRY loop [25,41–43]. Interestingly, several studies have pointed to the fact that the relevance of specific loops in rhythm generation might be tissue-dependent [44–46]. The growing pool of identified regulators for circadian oscillations and their corresponding feedback loops stress the fundamental importance of synergistic loops, that seem to confer robustness to the clock [35].

Experimental evidence has shown that expression of clock genes occurs via a regulated interaction of clock proteins with promoter regions of their target genes, namely D-boxes, REVERB/ROR-binding elements (RREs) and E-boxes, the so-called clock-controlled elements (CCEs) [43,47–49]. Binding of clock proteins to the different CCEs results in activation or repression of promoter elements, and consequently in the establishment of an intricate network of feedback loops [50–52]. Activation and repression of the distinct CCEs has been demonstrated to play an important role in the regulation of amplitude and transcriptional delay required for the generation of approximately 24 h oscillations [43]. Moreover, both timing and the order of regulation of such promoter elements seem to be critical for the phase of circadian oscillations [53].

Here, we address the question of whether the well-known coexistence of multiple feedback loops can generate complex nonlinear phenomena. We study data-driven models of gene-regulatory networks representing the mammalian circadian clock at the molecular level. As alterations in protein degradation rates are known to play a role in the oscillator's properties [41,54–56], we perform comprehensive bifurcation analyses to determine how changes in degradation rates affect oscillation dynamics. Using physiologically relevant parameters, we find that the interaction of multiple feedback loops can generate period-doubling, tori and deterministic chaos even in the absence of external forcing.

## 2. Results

### 2.1. Core-clock models exhibit multiple negative feedback loops

**Figure 2.**
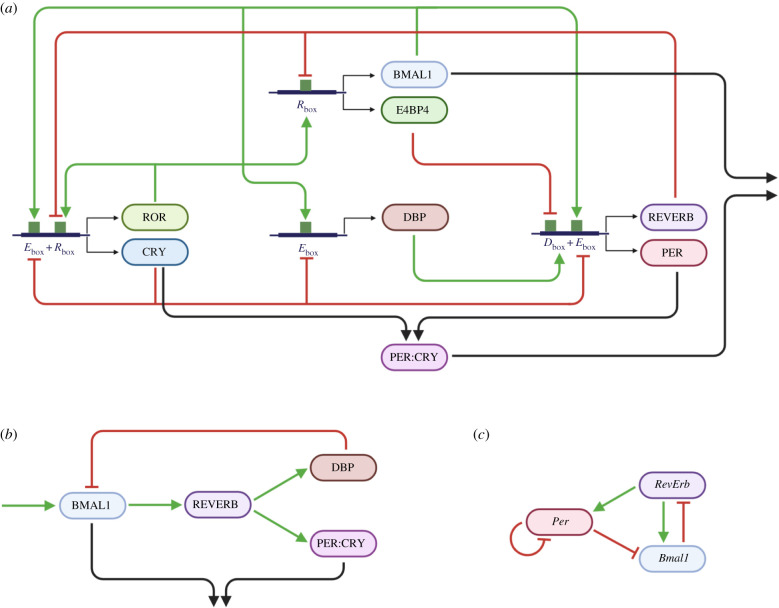
Circadian clock models exhibit multiple feedback loops. (*a*) Molecular mechanisms of the mammalian circadian clock in a schematic overview, as published by Almeida *et al*. [57]. (*b*) Simplification of the model in (*a*) to its core motif (details in appendix B). All names in (*a*) and (*b*) represent proteins, coloured arrows indicate positive (green) and negative (red) regulations of proteins on expression of other proteins. (*c*) Scheme of the transcription-based DDE model published by Schmal *et al.* [31], names represent different gene products. See the main text for details.

We chose recent models of the mammalian core clock of different mathematical structure and complexity that included at least two negative feedback loops as well as the core clock genes *BMAL1*, *PER* and *REVERB*. Figure 2*a* shows a protein-based model of the molecular mammalian circadian clockwork, including activation and inhibition of different CCEs. This model was developed by Almeida *et al.* [57] and is described by eight ordinary differential equations (ODEs) (appendix A) [57]. Here, BMAL1 (in complex with other proteins) drives the E-box dependent expression of clock genes, including *PER*, *CRY*, *REVERB*, *ROR* and *DBP.* CRY, alone and in complex with PER, inhibits BMAL1 activity (and other E-box containing genes) after a time delay [58,59]. At the same time, the BMAL1 and PER : CRY complexes can be inactivated and degraded [50,51]. Following translation, ROR and REVERB proteins compete to bind RREs at promoter regions of *BMAL1*. ROR acts as an activator and REVERB as an inhibitor. In short, the model in figure 2*a* contains three negative feedback loops, exerted by CRY, REVERB and PER : CRY on BMAL1, and one positive feedback loop mediated by ROR and BMAL1. Figure 2*b* shows a condensed representation of the model in figure 2*a*, where variables and feedback loops that are not required for rhythm generation are removed [25,57] (details of the model reduction are found in appendix B). The result is a four ODE model with only two negative feedback loops (BMAL1–REVERB and BMAL1–PER:CRY).

In order to validate whether nonlinear phenomena can arise in models of the molecular circadian clockwork, we took a third model of different complexity and mathematical structure. The model in figure 2*c* is a transcription-based model, displaying two negative feedback loops described in three delay differential equations (DDEs). This model is motivated by indications of an autonomous regulation of the *Per*-loop, independently of the *Bmal1*–*RevErb* loop [31]. Despite their differences, all models contain at least two negative feedback loops, long delays and nonlinearities, three features that are essential for rhythm generation [30,60,61].

### 2.2. Different circadian clock models exhibit self-sustained oscillations with a circadian period

**Figure 3.**
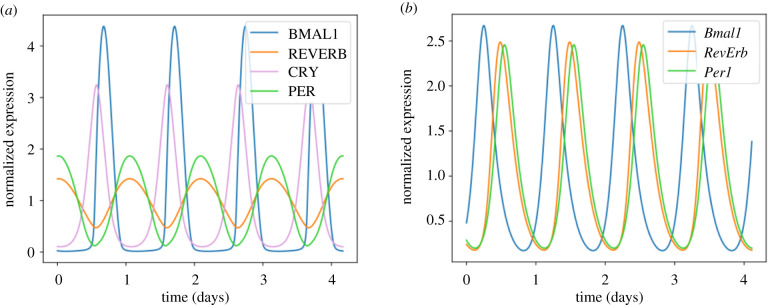
Different circadian clock models exhibit self-sustained oscillations with a circadian period. (*a*) Limit cycle oscillations obtained by numerical integration of the eight ODE model (equations shown in appendix A). The oscillation period is 24.8 h for the following parameter values: *V_R_* = 44.4 h^−1^, *k_R_* = 3.54, *k*_Rr_ = 80.1, *V_E_* = 30.3 h^−1^, *k_E_* = 214, *k*_Er_ = 1.24, *V_D_* = 202 h^−1^, *k_D_* = 5.32, *k*_Dr_ = 94.7, *γ*_ror_ = 2.55 h^−1^, *γ*_rev_ = 0.4 h^−1^, *γ_p_* = 0.844 h^−1^, *γ_c_* = 2.34 h^−1^, *γ*_db_ = 0.156 h^−1^, *γ*_E4_ = 0.295 h^−1^, *γ*_pc_ = 0.19 h^−1^, *γ*_cp_ = 0.141 h^−1^ and *γ*_bp_ = 2.58 h^−1^. (*b*) Limit cycle oscillations obtained by numerical integration of the three DDE model (equations shown in appendix A). The oscillation period is 24 h for the following parameter values: *d_P_* = 0.25 h^−1^, *d_B_* = 0.26 h^−1^, *d_R_* = 0.29 h^−1^, *v_P_* = 1, *v_B_* = 0.9, *v_R_* = 0.6, *k_P_* = 0.1, *k_B_* = 0.05, *k_R_* = 0.9, *c_P_* = 0.1, *c_R_* = 35, *b_P_* = 1, *b_R_* = 8, *T_P_* = 8.333 h, *T_R_* = 1.52 h and *T_B_* = 3.652 h. All time series are normalized to their means.

We numerically simulated the ODE models with the published parameter values [57], and performed control analysis on the eight ODE model to assess the effect of parameter changes on the oscillation period (appendix C). We found that the oscillation period strongly depended on the degradation rates of REVERB, E4BP4 and DBP (figure 10). Since the period of the ODE models was approximately 20 h [57], we tuned the degradation rate of REVERB (*γ*_REV_) to set the period to approximately 24 h. Simulations of the three DDE model with the published parameter set yielded 24 h oscillations [31]. The time series solutions of the eight ODE and three DDE models are shown in figure 3. The phase relationship between proteins (figure 3*a*) or transcripts (figure 3*b*) is in agreement with previous ChIP-Seq and proteomics results [53,62].

### 2.3. Bifurcation analyses reveal period-doubling and deterministic chaos in the complex ODE model but not in the reduced ODE model

Experimental and computational experiments have shown that alterations in clock protein and mRNA metabolism highly influence the oscillatory behaviour of the cellular circadian oscillator [22,63]. For example, familial advanced sleep phase syndrome (FASPS), a sleep disorder characterized by recurrent patterns of early evening sleepiness and early morning awakening, is associated with a mutation in PER2 that leads to its faster degradation and a shorter circadian period [54,64]. To analyse whether changes in degradation rates had an effect on the nonlinear behaviour of oscillations, bifurcation analyses were performed on all model parameters using continuation software [65].

**Figure 4.**
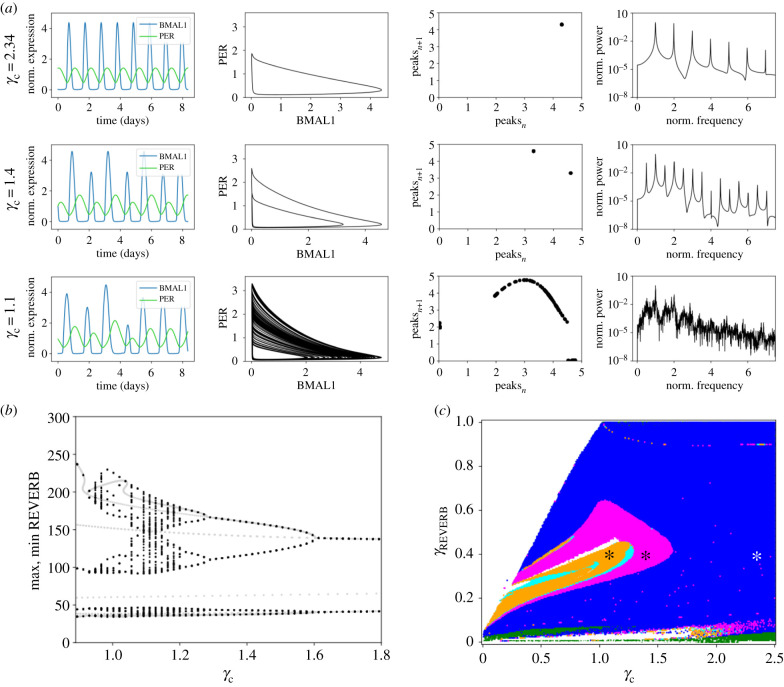
Bifurcation analyses reveal period-doubling and chaos in the eight ODE model. (*a*) Variations in *γ_c_* reveal nonlinear phenomena: limit cycle (top, *γ_c_* = 2.34 h^−1^), period-doubling (middle, *γ_c_* = 1.4 h^−1^), and chaos (bottom, *γ_c_* = 1.1 h^−1^) shown as time series, phase portraits, return maps and power spectra (from left to right, respectively). Parabola-shaped return maps are a feature of chaotic dynamics. The period in the power spectra is normalized to the period with maximum power. Note that chaotic oscillations were not normalized to obtain a 24 h peak-to-peak distance. Time series are normalized to their means. (*b*) Bifurcation diagram as a function of parameter *γ_c_.* Black dots represent the results of the brute force simulation, in which period-doubling cascades and chaos can be observed. Grey dots represent the results of the bifurcation analysis using continuation software. Note that absolute values of the simulated concentration of REVERB are shown here, instead of normalized values to the mean. (*c*) Two-dimensional bifurcation diagram as a function of parameters *γ_c_* and *γ*_REV_ reveals nonlinear phenomena: period-doubling (magenta), period quadrupling (cyan) and chaos (orange). Limit cycle is depicted in blue, stable steady state in green and white represents numerical instabilities. Asterisks indicate the parameter values from (*a*).

The bifurcation plots of the eight ODE model are shown in figure 11 (appendix D). Most of the parameter changes resulted in classical supercritical Hopf bifurcations, in which the system changed from a stable steady state into a stable limit cycle. However, changes in the degradation rate of CRY (*γ_c_*) revealed more complex nonlinear phenomena (figure 4, electronic supplementary material, video A1). CRY plays an essential role in the circadian clock: by itself, it represses E-boxes; and together with PER, it contributes to the inhibition of CLOCK : BMAL1 by a number of post-translational processes and nuclear export of the macromolecular complex [53,58,59]. Moreover, the differential role of homologues (CRY1, CRY2) as well as the severe arrhythmic phenotypes of CRY mutants stresses the biological relevance of CRY in the regulation of circadian oscillations [35,58]. This motivated us to analyse the effect of changes in *γ_c_* on the oscillatory behaviour of the model. The results in figure 4*a* show how the dynamics of the different model species change with *γ_c_*. We found period-doubling in the eight ODE model for *γ_c_* = 1.4 h^−1^ (middle panel), and chaos for *γ_c_* = 1.1 h^−1^ (bottom panel). We illustrate the dynamic behaviour in four different ways, namely as time series (first panels from left to right), as phase portraits (second panels), as return maps (third panels) [66] or as power spectra (right panels). Furthermore, a one-dimensional bifurcation diagram for *γ_c_* and two-dimensional bifurcation plot exploring the *γ_c_*–*γ*_REV_ parameter space are depicted in figure 4*b*,*c*, respectively. Figure 4*c* shows the regions in which nonlinear phenomena, namely period-doubling (magenta), period-quadrupling (cyan) and chaos (orange), occur.

**Figure 5.**
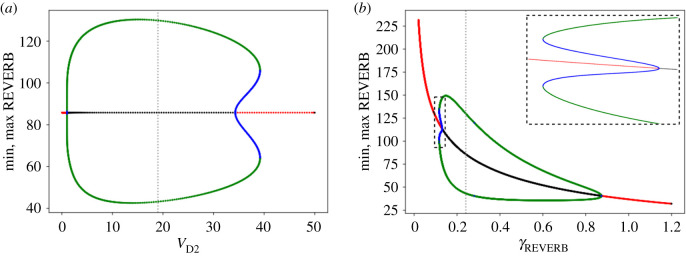
Nonlinear phenomena in the four ODE model. Examples of bifurcation diagrams of the model parameters *V_D2_* (*a*) and *γ*_REV_ (*b*) (equations and parameter values are provided in appendix B). The grey dashed lines indicate default parameter values. Continuation analysis revealed the onset of subcritical Hopf bifurcations, suggesting that limit cycle and steady-state coexist for a narrow parameter range around *V*_D2_ ∼ 34.29–39.21 h^−1^ (*a*) and *γ*_rev_ ∼ 0.12–0.13 h^−1^ (*b*). Bifurcation diagrams for the remaining model parameters are found in appendix F. Results are obtained with continuation software, diagrams show absolute values. The plots show stable steady state (red), unstable steady state (black), stable limit cycle (green) and unstable limit cycle (blue) solutions.

To determine if interlocked feedback loops are required for complex nonlinear phenomena to arise, we performed bifurcation analyses on the simplified four ODE model. To our surprise, and despite the similarities of the two ODE models, we did not observe period-doubling cascades or chaotic attractors (figure 5, figure 12, appendix E). Nevertheless, the oscillation onset via subcritical Hopf bifurcations when changing most of the parameters indicated that stable steady states and stable limit cycles coexist in certain parameter ranges (figure 12, appendix E). Taken together, our results suggest that the cooperating feedback loops of the eight ODE model are essential for the development of nonlinear phenomena.

### 2.4. Bifurcation analysis reveals that the dynamics of clock proteins can ‘split’ in the three DDE model

**Figure 6.**
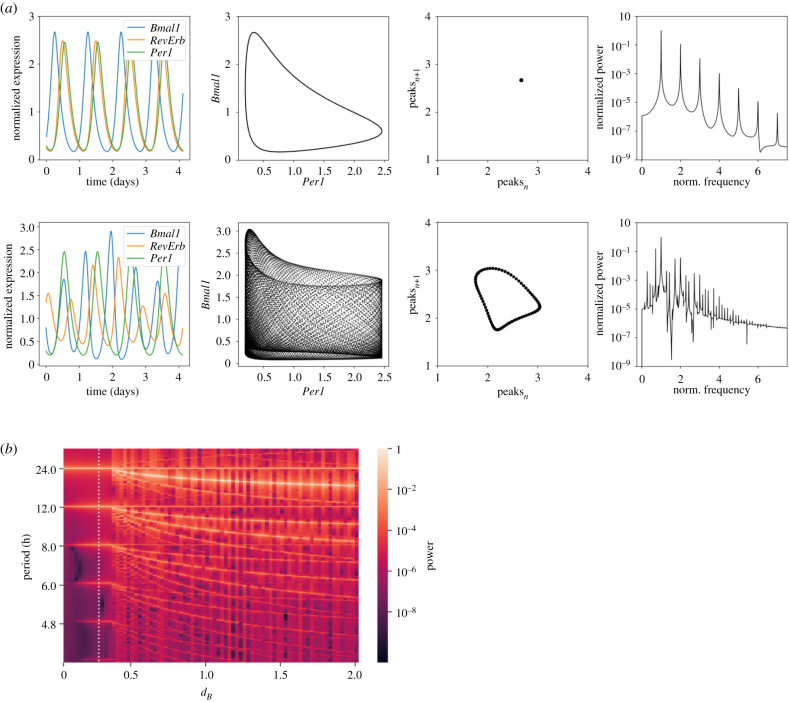
Bifurcation analyses reveal the existence of tori in the three DDE model. (*a*) Variations in *d_B_* reveal different nonlinear phenomena: limit cycle (top, *d_B_* = 0.26 h^−1^), and torus (bottom, *d_B_* = 1.5 h^−1^) shown as time series, phase portraits, return maps and power spectra (from left to right, respectively). Circular-shaped return maps are a feature of toroidal dynamics. The period in the power spectra is normalized to the period with maximum power. All simulated time series are normalized to their means. (*b*) Power spectral analysis indicating rhythm splitting of *Bmal1*. A period of 24 h (and harmonics) dominates the time series for *d_B_* < 0.36, but at this threshold, the dynamics split and other period values (with their respective harmonics) appear as well, resulting in toroidal dynamics of *Bmal1* (and *RevErb*, data not shown). *Per* oscillations remain with a 24 h rhythmicity (data not shown). Results are obtained by numerical integration of the equations as published by Schmal *et al*. [31]. White vertical line indicates the default parameter value. See the main text for details.

**Figure 10.**
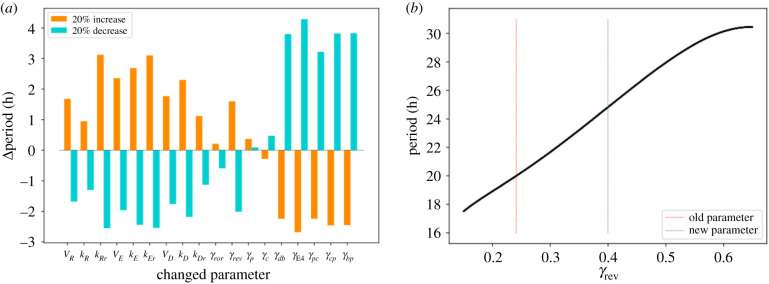
Control and sensitivity analyses for period tuning. (*a*) Period sensitivity analysis as a function of 20% increase (orange) or 20% decrease (blue) of the default parameter value as published by Almeida *et al*. [57]. The *y*-axis represents the difference between the period of BMAL1 oscillations calculated from the new parameter set compared to the default. Period values are obtained for each parameter set by numerical integration of the eight ODE model (figure 7). (*b*) Effect of change in *γ*_REV_ on the period. *γ*_REV_ was increased from 0.241 h^−1^ (red dashed line) to 0.4 h^−1^ (green dashed line) to set the period to approximately 24 h. After tuning, the parameters took the following values: *V_R_* = 44.4 h^−1^, *k_R_* = 3.54, *k*_Rr_ = 80.1, *V_E_* = 30.3 h^−1^, *k_E_* = 214, *k*_Er_ = 1.24, *V_D_* = 202 h^−1^, *k_D_* = 5.32, *k*_Dr_ = 94.7, *γ*_ror_ = 2.55 h^−1^, *γ*_rev_ = 0.4 h^−1^, *γ_p_* = 0.844 h^−1^, *γ_c_* = 2.34 h^−1^, *γ*_db_ = 0.156 h^−1^, *γ*_E4_ = 0.295 h^−1^, *γ*_pc_ = 0.191 h^−1^, *γ*_cp_ = 0.141 h^−1^ and *γ*_bp_ = 2.58 h^−1^.

In order to gain insights into how nonlinear phenomena might arise, and thus investigate whether such phenomena might be a common feature of circadian clock models or rather model-specific, we examined the simpler DDE model. Using comprehensive bifurcation and power spectral analyses, we studied how changes in parameters affected the dynamics of the three variables. The bifurcation analyses revealed the existence of robust tori upon variations in the degradation rates of *Bmal1* (*d_B_*), *RevErb* (*d_R_*) and upon changes in the rate of activation of *RevErb* expression (*k_R_*). The results of *d_B_* are shown in figure 6*a*, again as time series, phase portraits, return maps and power spectra, and in electronic supplementary material, video A2.

Toroidal dynamics are generated by two limit cycles of different frequencies. They are characterized by doughnut-shaped phase portraits and circular return maps [13]. Regarding power spectra, limit cycles can be distinguished from tori by the number of frequency peaks found in the spectrum. Whereas limit cycles show just one peak (and its subsequent harmonics), tori show more than one, which is why the literature also uses the term ‘splitting of periods' [33,67].

We illustrate that such period-splitting phenomenon can occur for *Bmal1* and *RevErb* dynamics in the DDE model in figure 6*b* and figure 13. In brief, figure 6*b* shows power spectra for numerical simulations which are run for different *d_B_* values (shown in the *x*-axis). Power spectra are colour coded, and thus, each vertical coloured line from the figure (i.e. each column) represents one power spectral density plot, for a certain parameter value. Below a certain parameter value threshold, variations in degradation rates of *Bmal1* (*d_B_*), *RevErb* (*d_R_*) and changes in *k_R_*, did not affect the robust rhythms, and the three variables oscillated with a 24 h period. However, when the threshold was reached (*d_B_* ∼ 0.36 in figure 6*b*), we observed the splitting of the dynamics. A second frequency, different to the 24 h period, appeared to dominate *RevErb* and *Bmal1* dynamics, resulting in the toroidal dynamics of the two variables. *Per* rhythms did not split, and thus *Per* kept displaying 24 h limit cycle oscillations (figure 6*b*). The splitting dynamics observed for *Bmal1 *and* RevErb* indicate that under some conditions (*d_B_* > 0.36, *d_R_* > 0.65 or *k_R_* > 1.38) the three DDE model can be ‘split’ into two individual oscillators, namely the autonomous *Per* oscillator and the *Bmal1-RevErb* oscillator (figure 2*c*).

## 3. Discussion

The presented results show that complex oscillatory phenomena can occur in realistic models of the circadian clock. Although chaotic dynamics were found to be theoretically possible in a model of the *Drosophila* clock [21], we have also found indications for such dynamic behaviour in the mammalian molecular circadian clock, even in the absence of external forcing. Nonlinear oscillatory phenomena, including chaos, are known to arise from the periodic forcing of a system. Indeed, chaos has been found in models that were forced by periodic light–dark cycles [30,32,34,35,68–70]. Here, in contrast, we focused on the case where nonlinear phenomena occur for circadian oscillations in constant conditions, in the absence of periodic stimuli.

It is known that single-loop negative feedback systems with a time delay can display limit cycle oscillations but do not exhibit chaos [14]. More complex dynamics, including period-doubling bifurcations leading to chaos, can appear in delayed negative systems with more than one negative feedback [71,72]. Although previous *Drosophila* clock models have reported chaotic dynamics [21,68] and, strictly speaking, contain only one negative feedback loop, one could argue in the lines of [73] that saturated degradation can be regarded as additional positive feedback loops. Thus, the number of loops increases and the complex nonlinear phenomena that appear are in agreement with theoretical predictions [14,71,72]. In our study, with comprehensive bifurcation analyses, we have detected robust tori in the two negative feedback loop DDE model and period-doubling cascades and chaos in the three negative feedback loop eight ODE model, supporting that multiple loops might be a common root of complex dynamic phenomena. Our results are of particular interest for biological systems with interlocked feedback loops, since we predict that tori, period-doubling cascades or chaotic dynamics might arise in such networks as well.

Bifurcation analyses on a manifold of clock models have shown that parameter changes can lead to transitions between stable equilibrium points and periodic limit cycle oscillations [41,74,75]. In this study, we have found that more complex bifurcations, such as subcritical Hopf bifurcations, period-doubling cascades that lead to chaos or bifurcations that result in toroidal oscillations, can occur in simple, yet comprehensive models of the mammalian circadian clock. Whereas arrhythmic behaviour at the molecular level usually arises from the deletion of clock genes [42,76], our results suggest that changes in a control parameter can also lead to transitions between periodic and chaotic oscillations.

**Figure 11.**
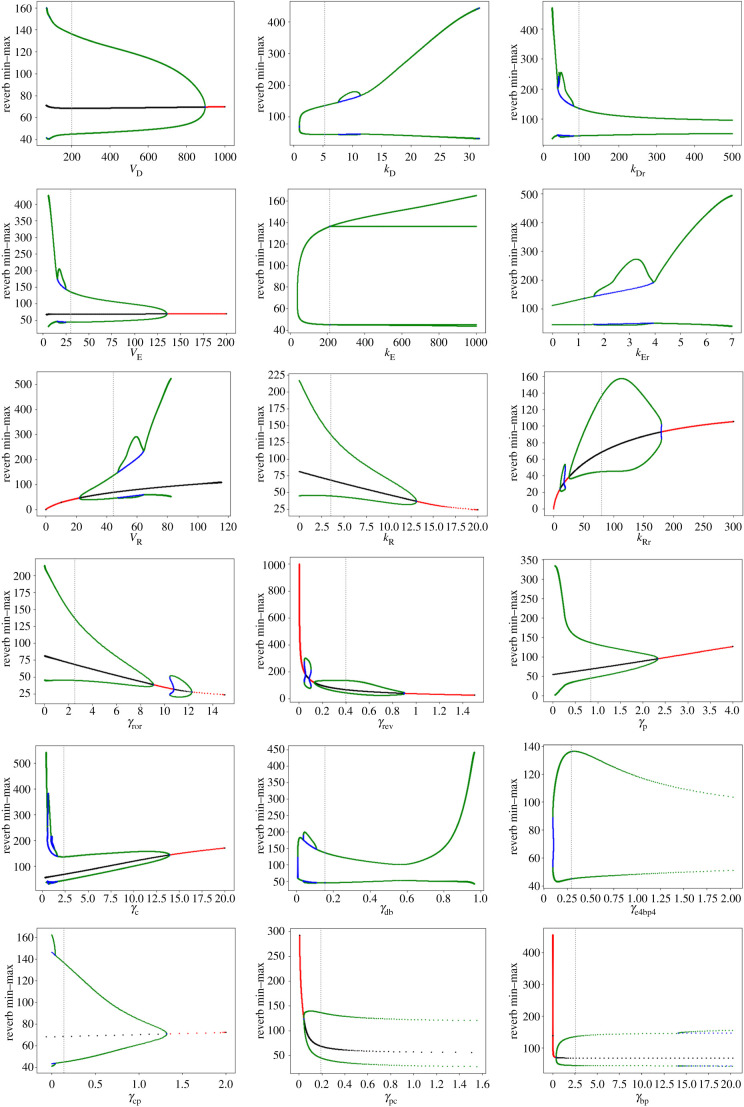
Bifurcation diagrams of the eight ODE model parameters, calculated using continuation software. Grey dashed lines indicate default parameters. The plots show stable steady state (red), unstable steady state (black), stable limit cycle (green) and unstable limit cycle (blue).

**Figure 12.**
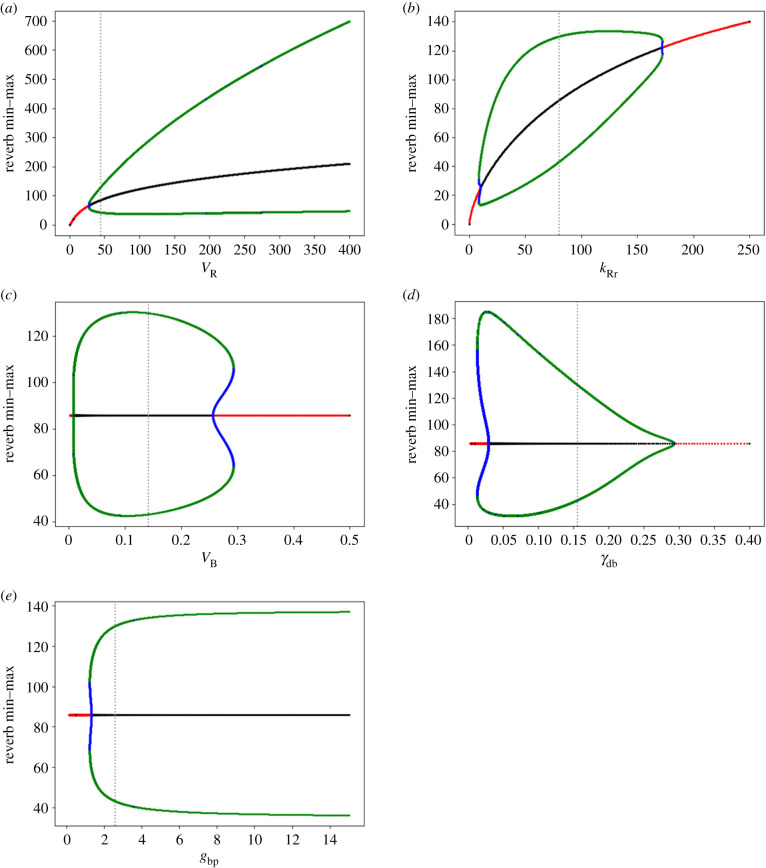
Bifurcation diagrams as a function of the four ODE model parameters *V_R_* (*a*), *k*_Rr_ (*b*), *V_B_* (*c*), *γ*_db_ (*d*) and *γ*_bp_ (*e*). Grey dashed lines indicate default parameters. The plots show stable steady state (red), unstable steady state (black), stable limit cycle (green) and unstable limit cycle (blue).

**Figure 13.**
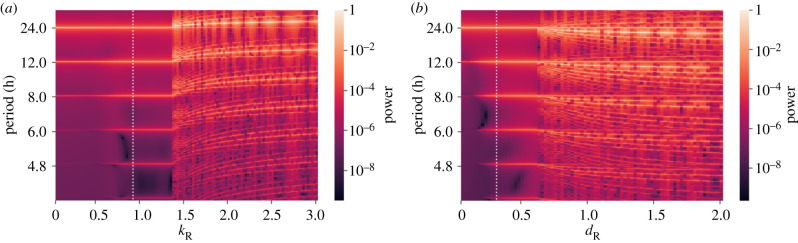
Power spectral analyses reveal period splitting of *Bmal1* dynamics upon changes in parameter values. In brief, the figures show power spectra for numerical simulations which are run for different *k_R_* (*a*) and *d_R_* (*b*) parameter values (shown on the *x*-axis). Power spectra are calculated for each parameter value and colour coded and thus, each vertical coloured line (i.e. column) from the figure represents one power spectral density plot, for a certain parameter value. Under a certain parameter value threshold, variations in *d_R_* and *k_R_* do not affect the robust 24 h period. However, when the threshold is reached (*k_R_* = 1.38, *d_R_* = 0.65), we observe splitting and a second frequency (and its harmonics) appears in the dynamics of *RevErb* and *Bmal1*, resulting in toroidal dynamics*. Per* oscillations remain with a 24 h rhythmicity (data not shown). Results are obtained by numerical integration of the equations as published by Schmal *et al*. [31]. The white vertical lines indicate default parameters.

Our results show that the cascade of period-doubling bifurcations leading to chaos is confined within a narrow domain in the *γ_c_* parameter space from the eight ODE model (figure 4*b* and figure 11). Toroidal oscillations, however, occur for a wider parameter range (figure 6*b* and figure 13). Tori are related to the phenomenon of rhythm splitting [77], which refers to the separation of two rhythms, of initially the same period, into two rhythms of markedly different periods. Such a frequency separation could be due to the operation of two different oscillators that progressively lose synchrony. It is thus tempting to speculate that the design principles of the three DDE model might be two oscillators (*Per* loop and *Bmal1–RevErb* loop) that over time drift apart and lose their 1 : 1 synchrony. Despite the relatively small size of the domains in parameter space in which complex oscillatory phenomena occur, the presence of chaos and splitting in the presented (realistic) models suggests that such phenomena might not be too uncommon in biological oscillatory systems, given that these systems are usually controlled by multiple mechanisms of cellular regulation.

Nonlinear phenomena in the absence of Zeitgeber stimuli have (experimentally) been shown to occur at the organismic level under certain conditions of circadian dysfunction. Some examples are the circadian desynchronization of organs and arrhythmic melatonin release [6,36]. However, the existence of such phenomena at the molecular level has not yet been confirmed. There have been some pioneering studies suggesting that rhythms of different reporter genes could dissociate in the SCN ([31,39,40], figure 1*b*), but the detailed mechanisms remain to be elucidated.

*In vivo* and *in vitro* degradation of clock proteins is described to be important in the regulation of circadian oscillations [41,54,78,79], as protein degradation rates modulate the length of the negative feedback loop needed for rhythm generation. For example, the PER2 mutation in FASPS is associated with a faster PER2 degradation and a shorter circadian period [54,63,80]. In addition, interfering with CRY degradation by knock-down of specific proteases results in longer periods [63]. Computational studies have also added to this picture, showing that changes in protein degradation rates can simulate knock-out and overexpression studies. This emphasizes the biological relevance of our bifurcation analyses [41]. A recent study by Pett *et al.* [45] suggested that different interlocked feedback loops might coexist and generate tissue-specific circadian rhythms. Taken together, the findings of potential tissue-specific clocks with the conditions under which nonlinear phenomena occur might contribute to the further understanding of organismic circadian desynchronization.

The interaction between changes in clock gene expression and the rise of arrhythmicities under some conditions illustrates the need for mathematical models to understand the underlying processes. Although a full representation of the biological systems is hard to reach due to modelling limitations, the presented nonlinear phenomena might help to understand how complex oscillatory dynamics occur at the molecular level and how the interactions result in arrhythmicities on the organismic level. However, experimental data will have to validate such nonlinear phenomena on the molecular level of the circadian clock *in vitro* and *in vivo*.

## 4. Material and methods

### 4.1. Model simulations and analyses

Temporal simulations and analyses from ODE models were performed in Python, using the odeint integrator from the scipy module; bifurcation analyses were done with the XPP-AUTO continuation software. Temporal simulations and analyses from the DDE model were performed in R, using the deSolve package. Computational results were stored and plotted with the matplotlib module from Python. Codes are available on request. Figure 1 was plotted with BioRender.

## Supplementary Material

Video A1

## Supplementary Material

Video A2 - A

## Supplementary Material

Video A2 - B

## Supplementary Material

Video A2 - C

## Figures and Tables

**Figure 7.**
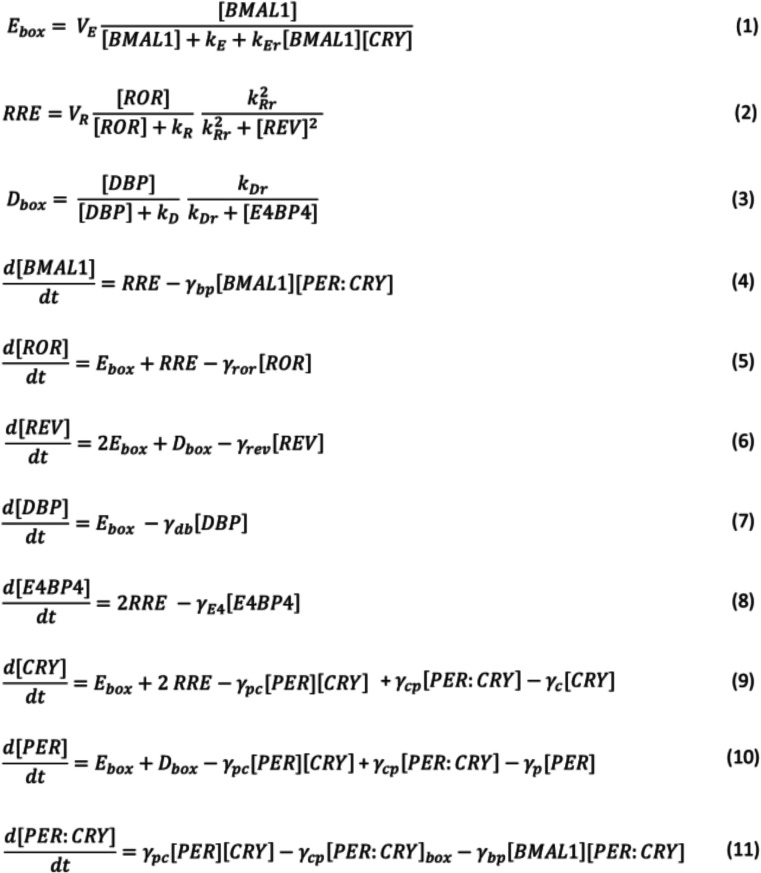
Equations of the eight ODE model, published by Almeida *et al*. [57]. Equations (1–3) describe activation/repression of the regulatory CCEs (E-box, RRE and D-box); equations (4–11) are the eight ODEs from the system, modelled with mass-action kinetics. Parameter values used for the numerical simulations are provided in figure 3*a*.

**Figure 8.**
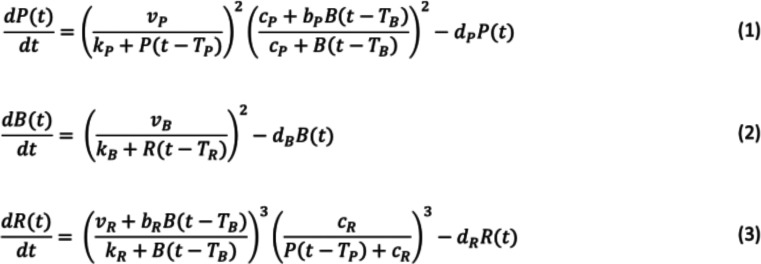
Equations of the three DDE model, published by Schmal *et al.* [31]. The equations describe the gene expression dynamics of *Per*, *Bmal1* and *RevErb* (equations 1–3), using delay-differential equations. Parameter values used for numerical simulations are provided in figure 3*b*.

**Figure 9.**
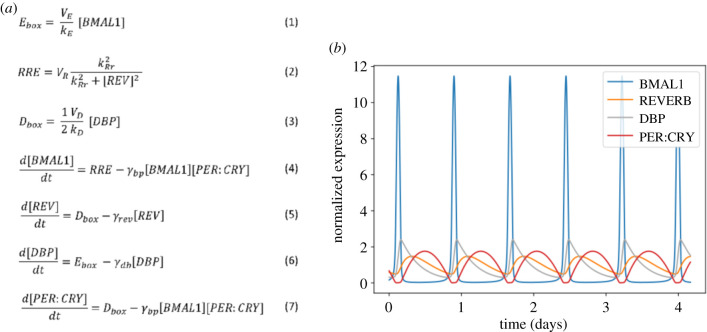
A simplified four ODE model for circadian oscillations. (*a*) Equations of the four ODE model including activation of CCEs (equations 1–3) and four ODEs describing the rate of change in protein concentration. Time series are normalized to their means. (*b*) Limit cycle oscillations obtained by numerical integration of the equations shown in (*a*) for the following parameter values: *V_R_* = 44.4 h^−1^, *k*_Rr_ = 80.1, *V_B_* = 0.142 h^−1^, *V*_D2_ = 19.0 h^−1^, *γ*_rev_ = 0.241 h^−1^, *γ*_db_ = 0.156 h^−1^ and *γ*_bp_ = 2.58 h^−1^.

## Data accessibility

All codes are available on request (marta.del-olmo@charite.de).

## Appendix A. Equations for the eight ODE and three DDE models

To investigate whether nonlinear phenomena appear in the mammalian molecular clock, we analysed two recent models of the mammalian circadian clockwork published by Almeida *et al*. [57] and Schmal *et al*. [31]. The model equations are shown in figures 7 and 8. In figure 7, the first three equations describe the regulation of the different CCEs (E-box, RRE and D-box), with their corresponding activators and repressors. Complex formation reactions, needed for the activation of promoter regions, are described by Michaelis–Menten-like terms and a Hill equation with an exponent of *n* = 2. Equations (4–11) describe the rates of change in protein concentrations with ODEs and mass action kinetics.

## Appendix B. Reduction of the eight ODE model to a four ODE model

In order to identify the proteins/model components that are necessary and sufficient to generate oscillations, we simulated the constitutive expression of the different proteins by assuming that their concentration did not change over time. Thus, we sequentially set the change in each protein over time to zero (quasi-steady state assumption, i.e. left-hand side of equations (4–11) from figure 7 equal to zero), a strategy that has been termed clamping [25,81]. If oscillations disappeared after clamping a variable, we concluded that the clamped variable was necessary for rhythm generation. If, on the other hand, the system still oscillated, we concluded that the clamped variable was not necessary for the generation of oscillations and subsequently removed the variable. By removing equations that were not essential for oscillations, the model was reduced from eight to four ODEs. The equations describing CCE regulation were accordingly modified by removing the pulled-out variables [82]. Equations of the simplified model and the corresponding oscillations are shown in figure 9.

## Appendix C. Period sensitivity analyses and tuning of the model

To analyse how sensitive the period of the eight ODE model was towards parameter changes, we increased and decreased all model parameters by 20% and quantified the effect of such change on the period of BMAL1 oscillations. The effect of changes in most of the degradation rates was opposite to the effect of changes in activating CCE parameters (figure 10*a*). The model published by Almeida *et al.* [57] showed oscillations with a period of approximately 20 h. Since the control analysis revealed that the period of the oscillations was relatively sensitive to changes in the degradation rate of REVERB (*γ*_REV_), we tuned the period of the oscillations to a more circadianly relevant value, namely 24.8 h, by changing *γ*_REV_ from 0.241 to 0.4 h^−1^ (figure 10*b*). The other parameter values remained unchanged.

## Appendix D. Bifurcation analyses of the eight ODE model

To investigate the presence of nonlinear phenomena in the eight ODE model, we performed one-dimensional bifurcation analyses for all model parameters. Most bifurcation diagrams displayed supercritical Hopf bifurcations, but nevertheless, changes in *γ_c_* (see main text) and *k*_Dr_ resulted in period-doubling cascades and chaotic dynamics. Results are shown in figure 11.

## Appendix E. Bifurcation analyses of the four ODE model

To examine whether all feedback loops of the eight ODE model were required for the development of complex nonlinear phenomena, we performed bifurcation analyses on the reduced four ODE model. We did not find neither chaos nor period-doubling, suggesting that interlocked feedback loops are required for these phenomena to emerge. Results are depicted in figure 12.

The onset of limit cycle oscillations occurred via subcritical Hopf bifurcations for many parameter changes, indicating that two attractors coexist for a narrow range of parameter values (*V_R_* ∼ 27.70–28.42 h^−1^, *k*_Rr_ ∼ 8.24–10.10 and 171.58–172.13, *V_B_* ∼ 0.26–0.29 h^−1^, *γ*_db_ ∼ 0.01–0.03 h^−1^ and *γ*_bp_ ∼ 0.12–0.13 h^−1^), namely limit cycle and stable steady state.

## Appendix F. Bifurcation analyses of the three DDE model

In order to investigate whether complex nonlinear phenomena were a common feature of circadian clock models or rather model-specific, we examined the presence of nonlinear phenomena on the three DDE model. Using period spectral analysis and three-dimensional-phase portraits, we investigated the effect of parameter changes on the period. Changes in *k_R_* and *d_R_* revealed that under a certain parameter value (*k_R_* < 1.38, *d_R_* < 0.65), 24 h rhythms dominate the time series of *Bmal1* and *RevErb* oscillations, as seen by the high power peak at 24 h and its harmonics (figure 13). Nevertheless, when these critical values are reached, period splitting occurs and toroidal oscillations arise (electronic supplementary material, video A2).
